# Anti-Inflammatory and Antioxidative *N*-Acetyldopamine Dimers from Adult *Vespa velutina auraria* Smith

**DOI:** 10.3390/molecules29225445

**Published:** 2024-11-19

**Authors:** Chao-He Liu, Xiu-Qing Pang, Qun Yu, Wei Zhang, Jing-Lei Xu, Yu-Chen Ma, Lei Huang, Geng Huang, Jia-Peng Wang, Huai Xiao, Zhong-Tao Ding

**Affiliations:** 1 Yunnan Provincial Key Laboratory of Entomological Biopharmaceutical R&D, College of Pharmacy, Dali University, Dali 671099, China; lch412728@163.com (C.-H.L.); p18787230348@163.com (X.-Q.P.); yqhmjk@163.com (Q.Y.); 15012318062@163.com (W.Z.); mayuchen95@163.com (Y.-C.M.); hl806333566@163.com (L.H.); hgeng20001223@163.com (G.H.); 2 Yunnan Yunke Characteristic Plant Extraction Laboratory Co., Ltd., Kunming 650106, China; 3 Key Laboratory of Luminescence Analysis and Molecular Sensing, Ministry of Education, College of Pharmaceutical Sciences, Southwest University, Chongqing 400715, China; xjl15093105827@163.com; 4 College of Traditional Chinese Medicine, Yunnan University of Chinese Medicine, Kunming 650091, China; ztding@ynu.edu.cn

**Keywords:** *Vespa velutina auraria* Smith, *N*-acetyldopamine dimers, veslumide, anti-inflammatory, antioxidant

## Abstract

One undescribed fatty glyceride (**1**), two unreported *N*-acetyldopamine dimers (**2** and **3**), and four known structurally diverse *N*-acetyldopamine dimers were isolated from adult *Vespa velutina auraria* Smith. Their structures were elucidated based on a comprehensive analysis of spectroscopic data, HRESIMS, and NMR calculations with ML_J_DP4, and the absolute configurations of **2** and **3** were determined via ECD calculations. Regarding their bioactivities, compounds **5** and **6** can inhibit the production of NO. Moreover, compounds **3**, **5** and **7** showed stronger antioxidant activity than the positive control (VC) at 14 μg/mL. A network pharmacology study was used to explore the potential bioactive mechanisms. In addition, a docking study of anti-inflammatory and antioxidative compounds was also performed.

## 1. Introduction

Insects dominate the earth in terms of both number of species and number of individuals. While the crucial role that insects play in the ecosystem has received great attention, studies on the chemical defense mechanisms used by insects only began in the late 1950s [1,2]. *Vespa velutina auraria* Smith (Hymenoptera Apocrita: Vespidae) is a species of wasp mainly found in Yunnan, Guizhou, and Sichuan, China. It is used to treat these diseases through the production of wasp wine [3,4], which is widely used among the Jingpo people, a Chinese national minority, and has been recorded in the Pharmacopoeia of the People’s Republic of China. *Vespa* was found to have positive economic impacts on beekeeping, agriculture, and human health [5]. *Vespa* also plays an important role in biological control by effectively managing numerous agricultural, forestry, and fruit pests such as *Helioithis armigera* Hubner, *Ostrinia mubilalis* Hubner, and *Leucania separata* Walker [6]. Moreover, in China and Korea, *Vespa* is not only valued as a food source due to its high content of amino acids and proteins but is also utilized for medicinal purposes to treat various ailments among the Jingpo people, a Chinese national minority, and has been recorded in the Pharmacopoeia of the People’s Republic of China [5,7]. A previous study has shown that wasps, bee venom, and honeycomb have a wide range of pharmacological effects, such as anti-cancer [8], antioxidant [9], anti-inflammatory, immunosuppressive [10], and anti-bacterial activities [11].

*N*-acetyldopamine (NADA) is a catecholamine derivative that is used by insects as a sclerotizing precursor to harden their cuticles [2,12]. It was first discovered in Calliphora-Larven [13], and has been reported in a variety of insects. Chemical investigations of some kinds of medicinal insects led to the isolation of novel *N*-acetyldopamine (NADA) derivatives, such as (±)-aspongamide A with anti-CKD activity [2], aspongdopamines that inhibit renal fibrosis [14], and percicamides with anti-inflammatory activity [15]. However, there are few reports studying the chemical composition of V. *velutina auraria*.

**Figure 1:**
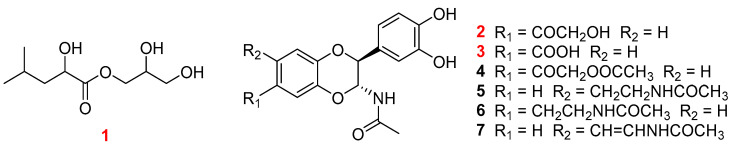
Structures of **1**–**7**.

Previously, we reported on the in vitro antioxidant and anti-inflammatory bioactivities of *V. velutina auraria* and its volatile composition, determined through gas chromatography mass spectrometry (GC-MS) [16]. The antioxidant activity screening results showed that the ethanol extracts of *V. velutina auraria* exhibited efficient antioxidant activity for three models. To provide ethnopharmacological evidence of the treatment effects of *V. velutina auraria*, an in-depth chemical and pharmacological investigation of the bioactive compounds of *V. velutina Auraria* was carried out. As a result (Figure 1), seven compounds, including one undescribed fatty glyceride (**1**), two undescribed NADA dimers (**2** and **3**), and four known compounds (**4**–**7**), were obtained (Figure 1). The ^1^H and ^13^C NMR data and the relative and absolute configurations of **2** were determined for the first time in this study. A discussion of their structural characterization and anti-inflammatory and antioxidative activities follows. Furthermore, network pharmacology and molecular docking were used to explore their potential anti-inflammatory and antioxidative mechanisms. For compounds **4**–**7**, this is the first report on their anti-inflammatory and antitumor activities.

## 2. Results

### 2.1. Structural Elucidation

Compounds **1** and **2** were obtained as a yellow powder mixture. The 2D NMR spectra showed no correlations between the two compounds, and distinct molecular ion peaks at *m*/*z* 229.1059 and 360.1106 were observed in the HRESIMS spectra. Although several isolated methods, including semi-HPLC, silica gel column chromatography and Sephadex column chromatography, were performed to isolate these two compounds, they still co-eluted in an approximately 1:1 ratio. This phenomenon might be due to the existence of intermolecular hydrogen bonding interactions between the two compounds [17].

**Figure 2:**
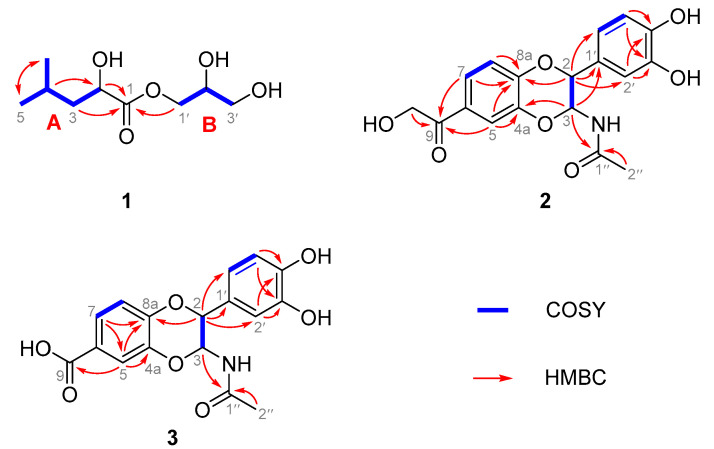
Key ^1^H-^1^H COSY and HMBC correlations of **1**–**3**.

Compound **1** has a molecular formula of C_9_H_18_O_5_, which was confirmed by the HRESIMS ion (Figure S6) at *m*/*z* 229.1059 ([M + Na]^+^, calculated for 229.1052); this implies one degree of unsaturation. An analysis of its ^1^H, ^13^C NMR (Table 1), and HSQC spectra (Figure S5) revealed the existence of nine carbon atoms, including two methyls [*δ*_H_ 0.98 (d, 6.6 Hz, H_3_-5), 0.96 (d, 6.5 Hz, H_3_-6); *δ*_C_ 23.9 (C-5), 22.3 (C-6)], three methylenes [*δ*_H_ 4.20 (m, H-1′a, oxygenated), 4.11 (dd, *J* = 11.3, 6.3 Hz, H-1′b, oxygenated), 3.55 (2H, d, *J* = 5.6 Hz, H-3′, oxygenated), 1.58 (2H, m, H-3); *δ*_C_ 44.4 (C-3), 67.1 (C-1′, oxygenated), 64.1 (C-3′, oxygenated)], three methines [*δ*_H_ 4.22 (m, H-2, oxygenated), 3.88–3.80 (m, H-2′, oxygenated), *δ*_H_ 1.87 (1H, m, H-4); *δ*_C_ 70.3 (C-2, oxygenated), 25.6 (C-4), 71.1 (C-2′, oxygenated)], and one carbonyl [*δ*_C_ 176.5 (C-1)]. ^1^H-^1^H COSY correlations (Figure 2 and Figure S3) of H-4 and H-2/H_2_-3, H-4/H_3_-5, H_3_-6 and H_2_-3, and HMBC correlations (Figure 2 and Figure S4) of H-4 and H_2_-3/C-1, indicated the presence of a 2-hydroxy-4-methylpentanoic acid unit (A). The chemical shifts in CH_2_-1, CH-2, and CH_2_-3 indicated that these three carbons are all bonded to oxygen atoms. These data, combined with the ^1^H-^1^H COSY correlations of H-2′/H_2_-1′ and H_2_-3′, demonstrated the presence of a glycerol unit (B). Finally, the HMBC correlation of H_2_-1′/C-1 suggested that two units (A and B) are linked by an ester bond, thus determining the planar structure to be a fatty glyceride. Because the pure example of **1** was not obtained, the relative and absolute configurations of **1** were not determined. Compound **1** was named 2′,3′-dihydroxypropyl-2-hydroxy-4-methylpentanoate.

**Figure 3:**
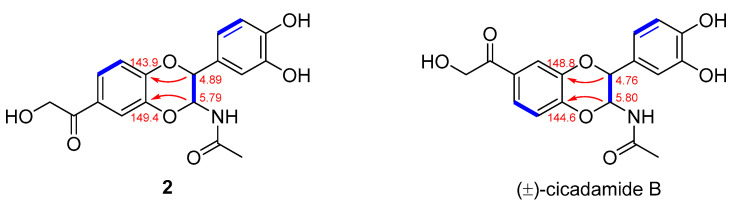
Key HMBC correlations and chemical shifts of C-3a and C-8a for **2** and (±)-cicadamide B.

The molecular formula of **2** was established as C_18_H_17_NO_7_ (eleven degrees of unsaturation) based on its HRESIMS [M + Na]^+^ ion peak at *m*/*z* 360.1106 (Figure S7) (calculated for 360.1083). Its 1D NMR data (Table 2) and HSQC spectra (Figure S5) revealed the existence of one methyl group [*δ*_H_ 1.91 (s, H-2″), *δ*_C_ 23.0 (C-2″)], one 1,2,4-trisubstituted aromatic ring [*δ*_H_ 7.55 (d, *J* = 2.0 Hz, H-5), 7.59 (dd, *J* = 8.4. 2.2 Hz, H-7), and 7.09 (d, *J* = 8.4 Hz, H-8); *δ*_C_ 143.9 (C-4a), 118.0 (C-5), 130.0 (C-6), 123.4 (C-7), 118.5 (C-8), and 149.4 (C-8a)], one 1,3,4-trisubstituted aromatic ring [6.88 (br s, H-2′), 6.80 (2H, overlap, H-4′ and 6′); *δ*_C_ 128.2 (C-1′), 115.9 (C-2′), 146.7 (C-3′), 147.5 (C-4′), 116.5 (C-5′), and 120.8 (C-6′)], one oxygenated methene [*δ*_H_ 4.85 (br s, H_2_-10, *δ*_C_ 66.4 (C-10)], two oxygenated methines [4.89 (d, *J* = 7.2 Hz, H-2), 5.79 (d, *J =* 7.2 Hz, H-3); *δ*_C_ 78.7 (C-2), 78.3 (C-3)], and two carbonyl carbons [*δ*_C_ 198.7 (C-9) and 172.9 (C-1″)]. These data suggest that compound **2** was an *N*-acetyl dopamine (NADA) derivative [18]. By comparing the spectroscopic properties with the reported data, although the ^13^C NMR data of compound **2** were almost the same as those of a known *N*-acetyldopamine dimer named (±)-cicadamide B [19], a detailed analysis of their ^1^H NMR data and HMBC correlations (Figure 2 and Figure 3) showed that compound **2** was different from (±)-cicadamide B. The main differences between **2** and (±)-cicadamide B are that the attributions of the chemical shift values of C-4a and C-8a are exactly opposite and the 2-hydroxyacetyl group (side chain) of **2** is located at C-6 (Figure 3), as confirmed by the ^1^H-^1^H COSY correlations (Figure 2 and Figure S3) of H-7/H-8 and HMBC correlations (Figure S4) of H-2/C-8a, H-3/C-4a, H-7/C-8a and C-9, H_2_-10/C-6 and C-9, and H-5/C-4a and C-9, as well as by the absence of HMBC correlations of H-7/C-4a. The HMBC correlations of H-3/C-1″ and H-2″/C-1” revealed the presence of an acetylamino group on C-3. In addition, the HMBC correlations of H-2/C-1′, C-2′, and C-6′ and H-3/C-1′ suggested that C-2 was attached to the C-1′ of 1,3,4-trisubstituted benzene ring. Thus, the planar structure of **2** was determined.

**Figure 4:**
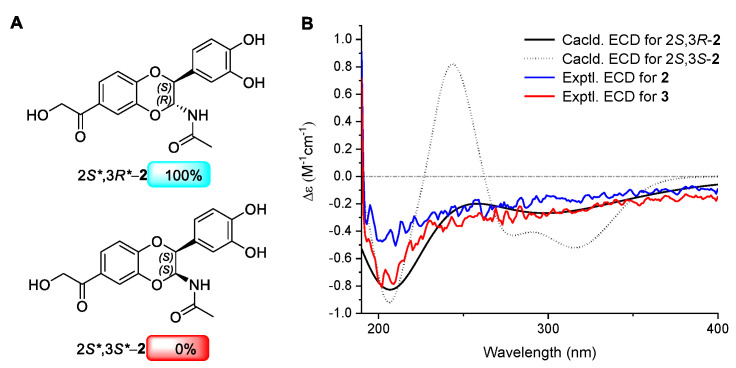
(**A**) ML_*J*_DP4 analysis of **2**. (**B**) ECD spectra of **2** and **3**.

The planar structure of **2** was previously reported by P. Roepstorff in 1981 [20]. However, its NMR data and relative and absolute configurations are still absent. Herein, we firstly determined its 1D and 2D NMR data and its relative and absolute configurations using NMR spectra, NMR calculations, and ECD spectra. The H-2/H-3 *o*-coupling constant *J* = 7.2 Hz for compound **2** indicated the existence of a *trans-*H-2/H-3 relationship [21,22], which allowed for the relative configuration of **2** as 2S*,3R* to be assigned. In addition, an ML_*J*_DP4 probability was determined to predict the probabilities of isomers 2S*,3R*- (*trans*) and 2S*,3S*-**2** (*cis*). NMR chemical shifts (GIAO) and ^3^*J*_H2-H3_ value were calculated at the rhf/sto-3g level, as required for ML_*J*_DP4 analysis [23]. The results unambiguously showed that 2S*,3R*-**2** was the most probable isomer, with 100% probability (Figure 4A and Figure S14). To clarify the absolute configuration of **2**, ECD calculations of 2*S*,3*R*- and 2*S*,3*S*-**2** at the B3LYP/6-311G//B3LYP/6-31G (methanol) level of theory were carried out [24,25]. It was found that the calculated ECD spectrum of 2*S*,3*R*-**2** agrees well with the experimental one (Figure 4B). This supports the results of the ML_*J*_DP4 analysis (Figure S14) and could also be used to determine the absolute configuration of **2** as 2*S*,3*R*. Compound **2** is therefore named veslumide A.

Compound **3** was obtained as a yellow powder. It showed a protonated molecular ion (Figure S13) at *m*/*z* 346.0943 ([M + Na]^+^, calculated for 346.0927) in the HRESIMS analysis, corresponding to the molecular formula C_17_H_15_NO_7_ (eleven degrees of unsaturation). A detailed analysis of its ^1^H and ^13^C NMR data (Table 2, Figures S8 and S9) indicated that the structure of **3** was similar to that of **2**, with the exception that one methylene group was absent. Combining the carbon chemical shifts of C-7 (*δ*_C_ 32.3) in **3** and the signal of a carboxyl (C-9) at 169.9 ppm from the HMBC spectrum (Figure S11) revealed that the side chain at C-7 in **2** was replaced with a carboxyl; this was further confirmed through the HMBC correlations of H-5 (*δ*_H_ 7.45, d, 1.8 Hz)/C-9. The *trans-*H-2 (*δ*_H_ 4.69 d, 7.2 Hz)/H-3 (*δ*_H_ 5.63, d, 7.2 Hz) relationship of **3** was determined via the *o*-coupling constant (*J* = 7.2 Hz), which was same as that of **2**. Finally, its ECD curves (Figure 4B), which were identical to those of **2,** showed the absolute configuration of **3** to be 2*S*, 3*R*. Thus, for compound **3**, the name veslumide B is proposed.

### 2.2. Assessment of Anti-Inflammatory Activity

**Figure 5:**
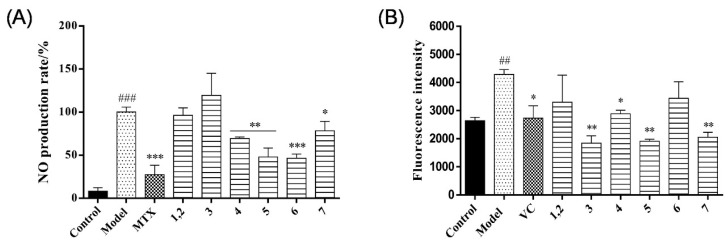
(**A**) Anti-inflammatory activity of all compounds on LPS combined with IFN-γ-induced RAW264.7 cells. * *p* < 0.05, ** *p* < 0.01, and *** *p* < 0.001 compared with model group. ### *p* < 0.001 compared with blank group. (**B**) Antioxidant activity of all compounds against H_2_O_2_-induced PC12 cells. * *p* < 0.05, and ** *p* < 0.01 compared with model group. ## *p* < 0.01 compared with control group. Data represent mean ± SEM values of the three experiments.

A previous study showed that NADA and its derivatives had anti-inflammatory and antioxidant activities [26]. Thus, the anti-inflammatory activity of all the isolates (30 μg/mL) and the positive drug methotrexate (30 μg/mL) was evaluated by examining the percentage of nitric oxide (NO) production induced by ipopolysaccharide (LPS) combined with interferon-γ (IFN-γ) in the mouse macrophage RAW264.7 cell line, as shown in Figure 5A. Compared with the model group, compounds **4**–**7** can moderately inhibit the production of NO. However, compounds **1**–**3** did not show significant anti-inflammatory effects. In addition, the effects of the antioxidant activity of the isolates on reactive oxygen species (ROS) production by PC12 cells were also evaluated. The concentration of isolates and positive drug vitamin C (VC) in the experiment was 14 μg/mL [27]. As shown in Figure 5B, compounds **1**–**7** were able to reduce the level of intracellular ROS to varying degrees. Notably, compounds **3**, **5**, and **7** showed greater antioxidant activity than the positive control (VC) at 14 μg/mL.

### 2.3. Molecular Docking Study

**Figure 6:**
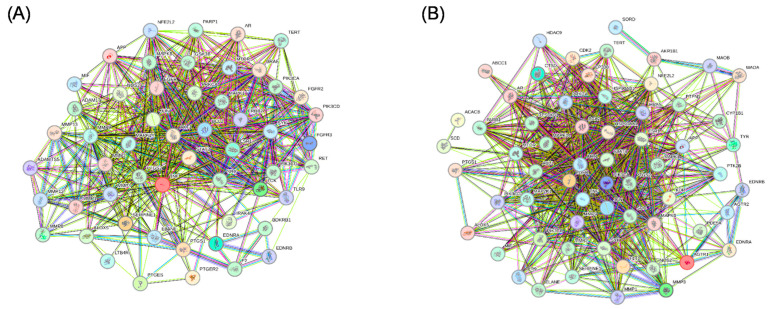
(**A**) The PPI network of 54 selected inflammatory targets. (**B**) The PPI network of 62 selected oxidative targets.

To further explore the anti-inflammatory and anti-oxidative stress mechanisms of the components from V. velutina auraria, the potential targets of seven isolated compounds were predicted using the Swiss Target Prediction database. Fifty-four anti-inflammatory targets and sixty-two anti-oxidative targets were obtained; the PPI network map was drawn using the STRING database (Figure 6A,B). Among the predicted anti-inflammatory targets, nitric oxide synthase (NOS2), a key target for the synthesis of NO, was involved in multiple inflammation-related pathways. NO is an intercellular communication substance involved in the regulation of pathophysiological processes such as vasodilatation and inflammatory immune response [28]. AKT1 is involved in oxidative stress-related pathways. It has been shown to protect osteoblasts from oxidative stress through the activation of the AKT-dependent NRF2 cascade [29].

Computational docking is an extremely useful in silico technique to obtain a deeper insight into drug–receptor interactions and plays a crucial role in the field of drug discovery. The more negative the estimated free binding energy (ΔG), the stronger the interactions between the ligand and the target enzyme and, consequently, the more stable the enzyme inhibitor complex that is formed. Bioactive compounds were individually docked to NOS2 (compounds **5** and **6**) and AKT1 (compounds **3**, **5**, and **7**) molecules and the binding energies were lower than −7 kcal/mol (Table 3 and Table 4), indicating a strong binding of the ligand to the receptor among the tested bioactive compounds. Compounds **3**, **5**, and **7** showed especially strong binding energies, with −9.4, −9.3, and −10.3 kcal·mol^−1^ for AKT1, indicating their potent antioxidative activity, which is in good agreement with the activity evaluation results.

**Figure 7:**
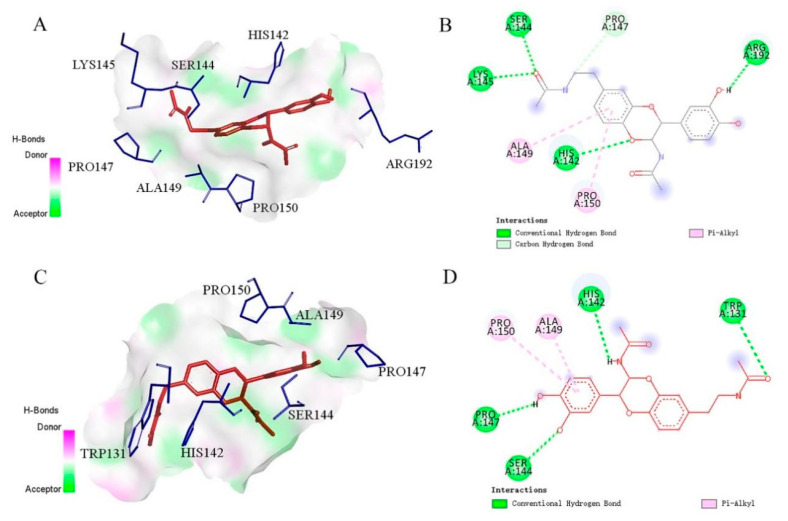
Molecular docking of anti-inflammatory compounds **5** (**A**,**B**) and **6** (**C**,**D**).

**Figure 8:**
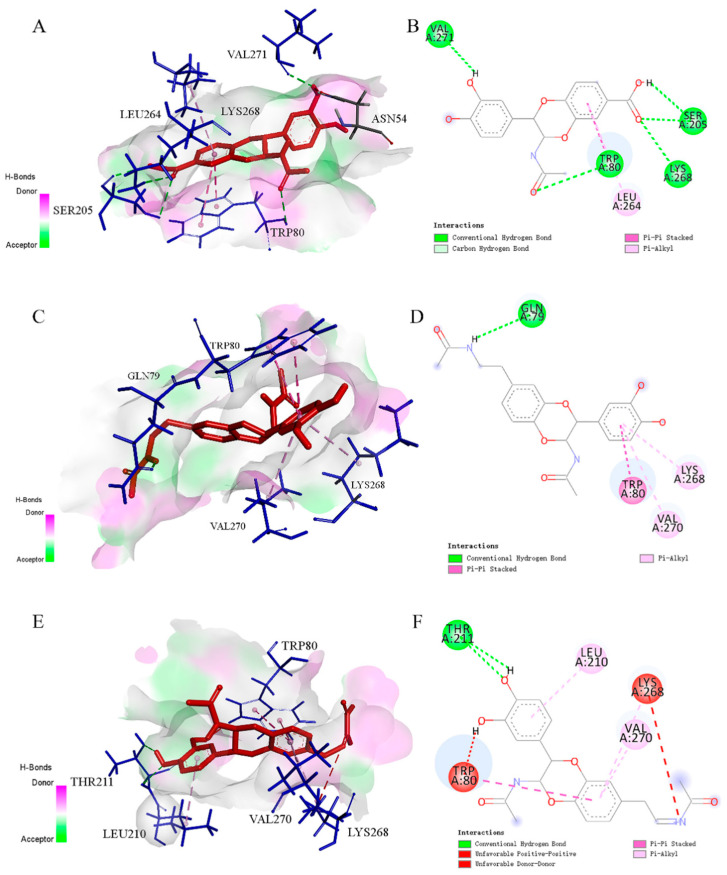
Molecular docking of anti-inflammatory compounds **3** (**A**,**B**), **5** (**C**,**D**), and **7** (**E**,**F**).

The molecular docking simulations of the anti-inflammatory results of bioactive compounds (**5** and **6**) in NOS2 (PDB: 4EY6) are presented in Figure 7. Overall, both compounds **5** and **6** can insert into the active pocket, interacting with the active site residues of the selected target protein. Compounds **5** and **6** formed several conventional hydrogen bonds with the Lys145, Ser144, His142, and Arg192 residues, and with the Pro147, Ser144, His142, and Trp131 residues, respectively. Moreover, the benzene ring of both **5** and **6** also participated in the *σ*-π stacking with the Ala149 and Pro150, indicating that an important role is played by the benzene ring. The results of molecular docking simulations of **3**, **5**, and **7** in AKT1 (PDB: 3O96) are shown in Figure 8. These three compounds formed several conventional hydrogen bonds or *σ*-π bonds with the Lys268, Trp 80, and Val271 residues. Moreover, the benzene rings of both **3** and **7** participated in the *σ*-π stacking with the Leu264 residue. Compound **5** also formed conventional hydrogen bonds with GLN79.

## 3. Materials and Methods

### 3.1. General Experimental Procedure

Separations were performed using a semi-preparative high-performance liquid phase (Reveleris^®^ PREP, Step Qi) with Sepax bio C18, XBridge peptide BEH C18 columns, and analyzed by HRESIMS on a Bruker Compact QTOF mass spectrometer (Bruker, Rheinstetten, Germany). All 1D and 2D NMR spectra (^1^H and^13^C NMR, ^1^H-^1^H COSY, HMBC, HSQC) were obtained on a Bruker AVANCE NEO NMR instrument (Bruker, Beijing, China). Separation and purification were performed using silica gel (200–300 mesh and 300–400 mesh, Qingdao Ocean Chemical Co., Ltd., Qingdao, China), dextran gel Sephadex LH-20 (GE Healthcare, Pittsburgh, PA, USA), and thin-layer chromatography on GF254 thin-layer chromatography silica gel plates (Qingdao Ocean Chemical Co., Ltd., Qingdao, China). The organic solvents used for sample extraction, column chromatography, preparative HPLC, and thin-layer chromatography were of analytical grade, with methanol (MeOH, Sichuan Xilong Science Co., Ltd., Chengdu, China) used for semi-preparative HPLC.

### 3.2. Insect Material

*Vespa velutina auraria* Smith (Vespidae) was provided by the Vespa Breeding Base in Dehong Prefecture, Yunnan Province, China (coordinates 24.5 N, 98.5 E) in November, winter, 2019. Professor Zi-Zhong Yang of Dali University identified it as *Vespa velutina auraria* Smith, and the specimen (No. 20191109001) was preserved in Yunnan Provincial Key Laboratory of Entomological Biopharmaceutical R&D, College of Pharmacy, Dali University.

### 3.3. Extraction and Isolation

A total of 32 kg of dry powder of the adult of *Vespa velutina auraria* was soaked in 95% ethanol solution for 3 days, mixing once a day at a ratio of 1:3. After cold soak extraction, the soaking solution was filtered through a 400 mesh filter cloth, the filtrate was extracted, and the filtrate was collected and evaporated under reduced pressure. The crude residue (0.8 kg) was suspended in H_2_O and partitioned three times into petroleum ether (PE, A), chloroform (CHCl_3_, B), and ethyl acetate (EtOAc, C) portions.

The chloroform-soluble fraction B (39.0 g) was separated using column chromatography on silica gel, eluting stepwise with CHCl_3_: MeOH (50:1, 25:1, 10:1, 5:1, 2:1, 1:1, *v*/*v*) to create fractions B1~B5. B5 (4.5 g) was subjected to 200–300 mesh silica gel column chromatography with CHCl_3_: MeOH (25:1→5:1, *v*/*v*) gradient elution and further purified by Sephadex LH-20 gel elution (MeOH) to obtain compound **5** (22.1 mg). Fraction C (92 g) was separated using silica gel column chromatography with a gradient elution of CHCl_3_: MeOH (25:1, 10:1, 5:1, 3:2, 0:1, *v*/*v*) to obtain fractions C1~C11. Fraction C5 was separated using silica gel column chromatography with a gradient elution of CHCl_3_: MeOH (5:1→1:1, *v*/*v*) and continuously purified via silica gel column chromatography and Sephadex LH-20 gel to obtain compound **3** (9.7 mg) and fraction C5-1. Then, C5-1 was separated using pre-HPLC (flow rate: 17 mL/min, MeOH: H_2_O = 30:30→60:30) to create compound **4** (18 mg, t_R_ = 17 min). C6 was separated using pre-HPLC (flow rate: 2 mL/min, MeOH: H_2_O = 5: 95) to obtain a mixture of compounds **1** (9.6 mg) and **2** (10.3 mg). C7 was separated by pre-HPLC (flow rate: 17 mL/min, MeOH: H_2_O = 5:95→95:5) and purified by Sephadex LH-20 gel (MeOH) to obtain compounds **6** (93.6 mg) and **7** (26.2 mg).

Mixtures (**1** and **2**): yellow amorphous powder; [*α*${]}_{D}^{20}$ +140.0 (c 0.07, MeOH), ^1^H NMR (400 MHz, CD_3_OD), and ^13^C NMR (100 MHz, CD_3_OD); see Table 1 and Table 2; positive HRESIMS *m/z*: compound **1**, 229.1059 ([M + Na]^+^, calculated for 229.1052); compound **2**, 360.1105 (calculated for 360.1083).

Veslumide B (**3**): yellow amorphous powder; [*α*${]}_{D}^{20}$ +332.0 (c 0.17, MeOH), ^1^H NMR (600 MHz, CD_3_OD) and ^13^C NMR (150 MHz, CD_3_OD); see Table 2; positive HRESIMS *m/z*: 346.0943 ([M + H]^+^, calculated for 346.0921).

### 3.4. NMR Computational Methods

The theoretical calculations of compound **2** were performed using the Gaussian 09 package. All conformers found at the MMFF level (energy cutoff of 5.0 kcal/mol) were submitted to GIAO NMR calculations at the hf/sto-3g level (using the pop = nbo option) [23]. The corresponding Gaussian output files were then fed to the ML-*J*-DP4 Python module, which can be easily installed via console using pip3 install ml-jdp4. The program creates an input matrix by computing different local descriptors from the 3D geometries and NMR/NBO data. The input matrix is transformed into refined chemical shifts using a KRR-trained ML. The chemical shifts and coupling constants are automatically Boltzmann-averaged and correlated with the experimental data provided to obtain the ML-*J*-DP4 probabilities for each candidate isomer. A detailed step-by-step user guide is available at https://github.com/Sarotti-Lab/ML_J_DP4, accessed on 13 September 2023.

### 3.5. TDDFT-ECD Calculations

The theoretical calculations of compound **2** were performed using the Gaussian 09 package. Conformational searches for **2** were performed via molecular mechanics using the MMFF method. The geometries were further optimized at the B3LYP/6-311+G level using Gaussian 09 software to provide the energy-minimized conformers. Then, the optimized conformers were subjected to the calculations of ECD spectra using TDDFT at the B3LYP/31G (d,p) level; solvent effects of the methanol solution were evaluated at the same DFT level using the polarizable continuum model (PCM) method. The ECD spectra of **2** were obtained by weighing the Boltzmann distribution rate of each geometric conformation [24].

### 3.6. Cell Culture and Cellular Anti-Inflammatory Assay

The mouse monocyte macrophage leukemia cell line RAW264.7 from Wuhan Purosai Life Sciences Co., Ltd., was preserved in Dulbecco’s modified Eagle’s medium (Purosai, Hubei, China) and the culture medium was supplemented with 1% penicillin mixture (Solabao, Beijing, China) and 10% fetal bovine serum (GIBCO, Waltham, CA, USA) in an incubator at 37 °C and 5% CO_2_. The anti-inflammatory levels of the compounds were evaluated by measuring the levels of NO in the cell supernatants using the Griess method [30]. Cells were inoculated into 96-well plates at a density of 1.0 × 10^5^ cells/mL and treated with a combination of LPS (1 μg/mL) and IFN-γ (20 ng/mL) for 24 h, followed by incubation with methotrexate and compounds (30 μg/mL) for 24 h. Finally, 100 μL of the cell supernatant was added to 100 μL of Griess Reagent to detect the NO content. The optical density at 540 nm was measured using an enzyme marker. The percentage of NO production was the NO concentration in each group/NO concentration in the model group.

### 3.7. Anti-Oxidative Activity

DCFH-DA fluorescent labeling was used to detect the level of ROS in PC12 cells, and the degree of intracellular oxidative stress was reflected by the change in ROS level [31]. PC12 cells were seeded in a 96-well plate at 1 × 10^4^ cells per well and cultured at 37 °C and 5% CO_2_ for 24 h. The cell survival rate of H_2_O_2_ at different concentrations (100, 200, 400, 600, and 800 μM) and stimulation times (0, 1, 2, 3, and 4 h) [32] was measured using the MTT method. According to the manufacturer’s plan, intracellular ROS levels were measured using an ROS assay kit. In short, PC12 cells were treated with 1.5 mL DCFH-DA (10 μM), and incubated at room temperature for 20 min. Then, the cells were washed and collected, and the fluorescence intensity of each ROS group was detected using a multi-mode reader at excitation/emission wavelengths of 488/525 nm. The mean fluorescence intensity was quantitatively analyzed.

### 3.8. Network Pharmacological Analysis

In this study, the 2D structures of the compounds were first constructed using the Pubchem database; then, the SMILES numbers of the compounds were imported into the database Swiss Target Prediction to predict the targets of compound action. Then, the GeneCards (https://www.genecards.org/, accessed on 18 September 2023) were used to retrieve inflammatory targets [33], and after screening, the intersection was taken using the Wayne diagram to obtain the disease targets that could be analyzed. This was followed by protein–protein interaction analysis using the STRING database (https://string-db.org/, accessed on 18 September 2023) [34], and Cytoscape v3.9. software for the visualization of the results.

### 3.9. Docking Study

Molecular docking with the protein crystals corresponding to the screened core targets was performed using the software Autodock 4.2.6. The pdb files of the NOS2 (PDB: 4EY6) and AKT1 (PDB: 3O96) were retrieved from the Brookhaven protein database https://www.rcsb.org/structure/2WJO, accessed on 25 September 2023. Subsequently, water molecules and the cognate ligand were removed from the receptor using Discovery Studio Visualizer. The protein was converted to the required pdbqt format using the Autodock Tools package (1.5.6rc). The structures of compounds **3**, **5**, and **7** were sketched and saved in pdb format using Marvin Sketch 15.6.8 www.chemaxon.com, accessed on 25 September 2023. The ligand molecule was saved in pdbqt format after adding Kollman charges. The pdbqt file of the ligands was prepared using Auto Dock tools (1.5.6rc). Docking simulations were performed with the Autodock tools (1.5.6rc) within a docking box. At the end of the docking simulations, the best docking solutions were selected for further analysis of enzyme–inhibitor interactions. The poses with the lowest binding score and most favorable affinity were selected and visualized using Discovery Studio Visualizer.

## 4. Conclusions

In summary, three unreported natural compounds and four known dopamine derivatives were isolated from the adult of *V. velutina auraria*. The structures of the new compounds were identified through a combination of HRESIMS, NMR spectroscopy, NMR calculations, and ECD. In vitro, compounds **4**–**7** exhibited anti-inflammatory activity against the mouse monocyte-macrophage cell line RAW264.7. Compounds **3**, **5,** and **7** also showed potent antioxidant activity. Network pharmacology studies and docking studies were performed to predict their potential bioactive mechanism. This research presents the promising anti-inflammatory and antioxidant activities of natural products for further pharmacological investigation. This study also provides evidence for further research on and utilization of *V. velutina auraria*.

## Figures and Tables

**Table 1:** ^1^H (400 MHz) and ^13^C (100 MHz) NMR data of **1** (recorded in CD_3_OD, *δ* in ppm).

No.	1	
	*δ*_H_ (*J* in Hz)	*δ*_C_
1		176.5
2	4.22 (dd, 5.6, 2.8)	70.3
3	1.58 (2H, m)	44.4
4	1.87 (m)	25.6
5	0.98 (d, 6.8)	23.9
6	0.96 (d, 6.8)	22.3
1′a 1′b	4.20 (d, 4.0) 4.11 (dd, 11.6, 6.4)	67.1
2′	3.84 (m)	71.1
3′	3.55 (d, 5.6)	64.1

**Table 2:** ^1^H (400 MHz) and ^13^C (100 MHz) NMR data of **2** and **3** (recorded in CD_3_OD, *δ* in ppm).

No.	2		3	
	*δ*_H_ (*J* in Hz)	*δ*_C_	*δ*_H_ (*J* in Hz)	*δ*_C_
1				
2	4.89 (d, 7.2)	78.7	4.69 (d, 7.2)	78.7
3	5.79 (d, 7.2)	78.3	5.63 (d, 7.2)	78.3
4				
4a		143.9		143.4
5	7.55 (d, 2.0)	118.0	7.45 (d, 1.8)	119.8
6		130.0		126.2
7	7.59 (dd, 8.4, 2.2)	123.4	7.48 (dd, 8.4, 2.4)	124.9
8	7.09 (d, 8.4)	118.5	6.89 (d, 8.4)	117.8
8a		149.4		148.5
9		198.7		169.9
10	4.85 (2H, s)	66.4		
1′		128.2		128.2
2′	6.88 (brs)	115.9	6.75 (d, 1.2)	115.6
3′		146.7		146.5
4′		147.5		147.4
5′	6.80 (overlap)	116.5	6.67 (overlap)	116.2
6′	6.80 (overlap)	120.8	6.67 (overlap)	120.7
1″		172.9		173.3
2″	1.91 (s)	23.0	1.79 (s)	22.6

**Table 3:** Docking binding energy of compounds **5** and **6** with NOS2.

Compound	Binding Energy (kcal·mol^−1^)
**5**	−7.1
**6**	−7.0

**Table 4:** Docking binding energy of compounds **3**, **5**, and **7** with AKT1.

Compound	Binding Energy (kcal·mol^−1^)
**3**	−9.4
**5**	−9.3
**7**	−10.3

## Data Availability

Upon request to the corresponding authors.

## Supplementary Materials

The following supporting information can be downloaded at: https://www.mdpi.com/article/10.3390/molecules29225445/s1, Figures S1–S13: MS and NMR spectra of compounds **1**–**3**; Figure S14: ML_J_DP4 results of compound **2**.
