# Supplementary material for: Anti-Inflammatory and Antioxidative N-Acetyldopamine Dimers from Adult Vespa velutina auraria Smith
Source: Molecules. 2024 Nov 19;29(22):5445. doi: 10.3390/molecules29225445 (PMC11597435; doi:10.3390/molecules29225445)
Supplement: Supplementary file 1 [file molecules-29-05445-s001.zip › molecules-3248290-supplementary.pdf]

Table of Contents

Figure S1. ^{1}H NMR spectrum ($CDCl_3$, 400 MHz) of 1 and 2 3

Figure S2. ^{13}C and DEPT spectra ($CDCl_3$, 100 MHz) of 1 and 2 4

Figure S3. ^{1}H-^{1}H COSY spectrum ($CDCl_3$, 400 MHz) of 1 and 2 5

Figure S4. HMBC spectrum ($CDCl_3$, 400 MHz) of 1 and 2................ 6

Figure S5. HSQC spectrum ($CDCl_3$, 400 MHz) of 1 and 2................ 7

Figure S6. (+)-HR-ESI-MS (positive mode) of 1................ 8

Figure S7. (+)-HR-ESI-MS (positive mode) of 2................ 8

Figure S8. ^{1}H NMR spectrum (MeOD, 600 MHz) of 3 9

Figure S9. ^{13}C and DEPT spectra (MeOD, 150 MHz) of 3 10

Figure S10. ^{1}H-^{1}H COSY spectrum (MeOD, 600 MHz) of 3 11

Figure S11. HMBC spectrum (MeOD, 600 MHz) of 3................ 12

Figure S12. HSQC spectrum (MeOD, 600 MHz) of 3................ 13

Figure S13. (+)-HR-ESI-MS (positive mode) of 3................ 14

Figure S14. ML-JDP4 results of 2*S**,3*S**-2 (Isomer 1) and 2*S**,3*R**-2 (Isomer 2). 15

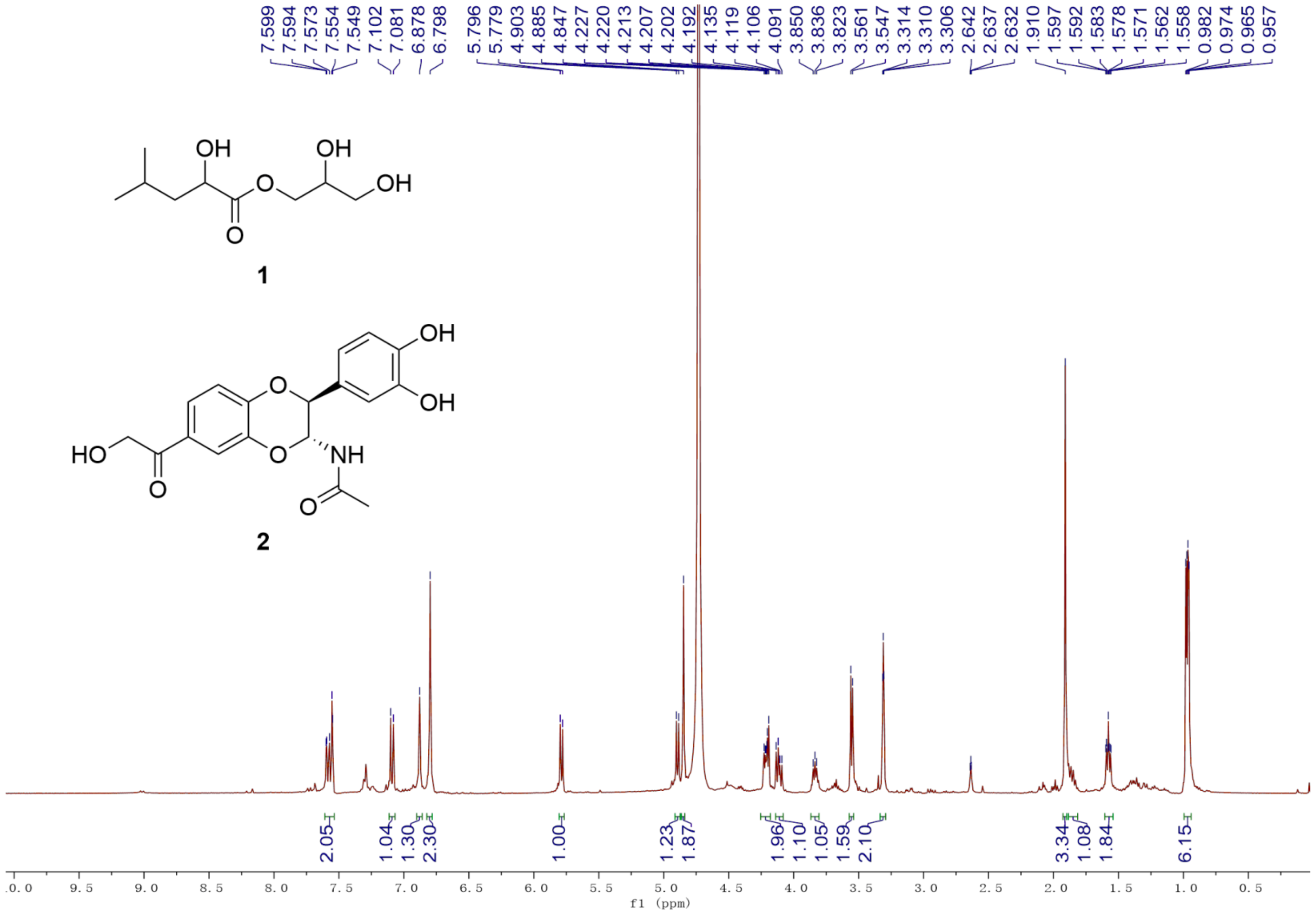

Figure S1. ^{1}H NMR spectrum ($CDCl_3$, 400 MHz) of **1** and **2**

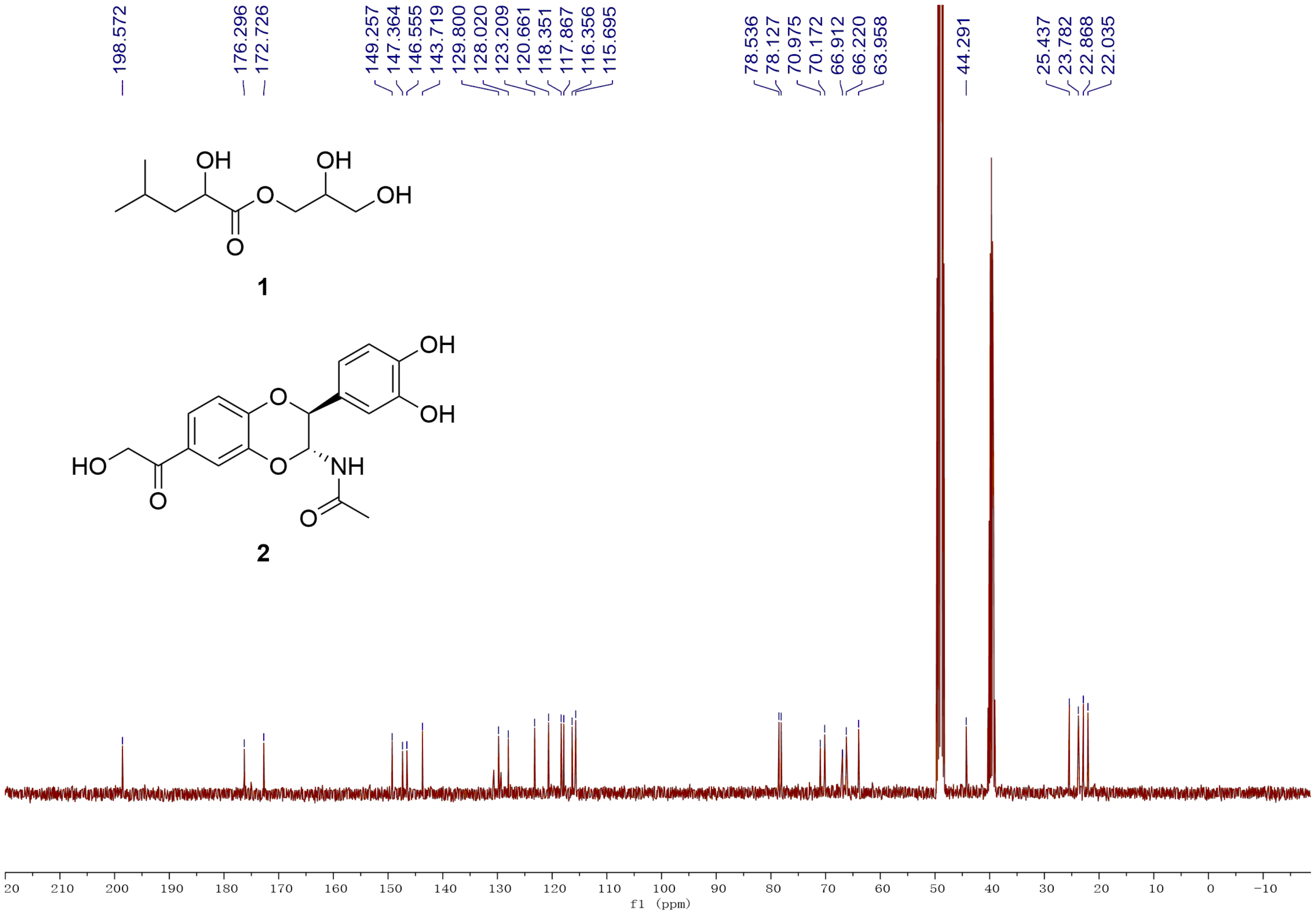

Figure S2. ^{13}C and DEPT spectra ($CDCl_3$, 100 MHz) of **1** and **2**

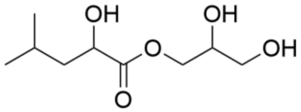

1

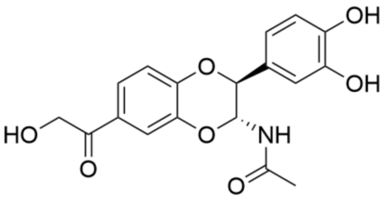

2

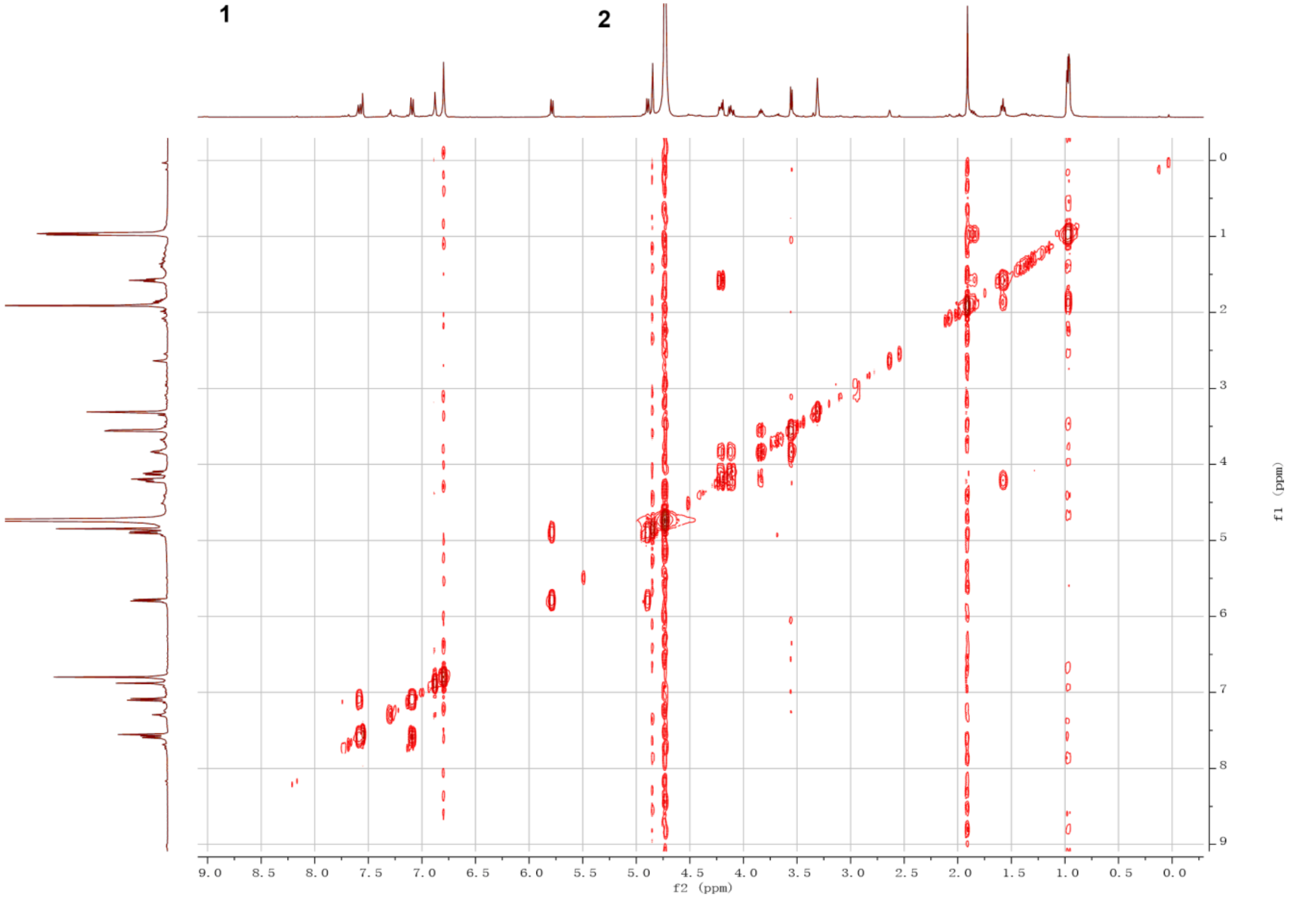

Figure S3. ^{1}H-^{1}H COSY spectrum ($CDCl_3$, 400 MHz) of **1** and **2**

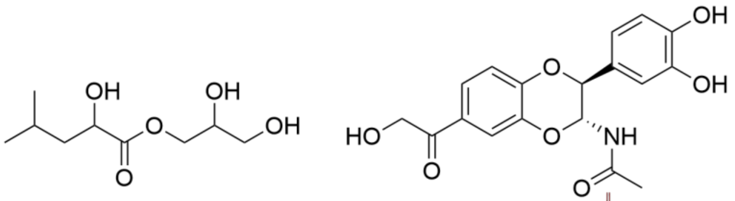

1 **2**

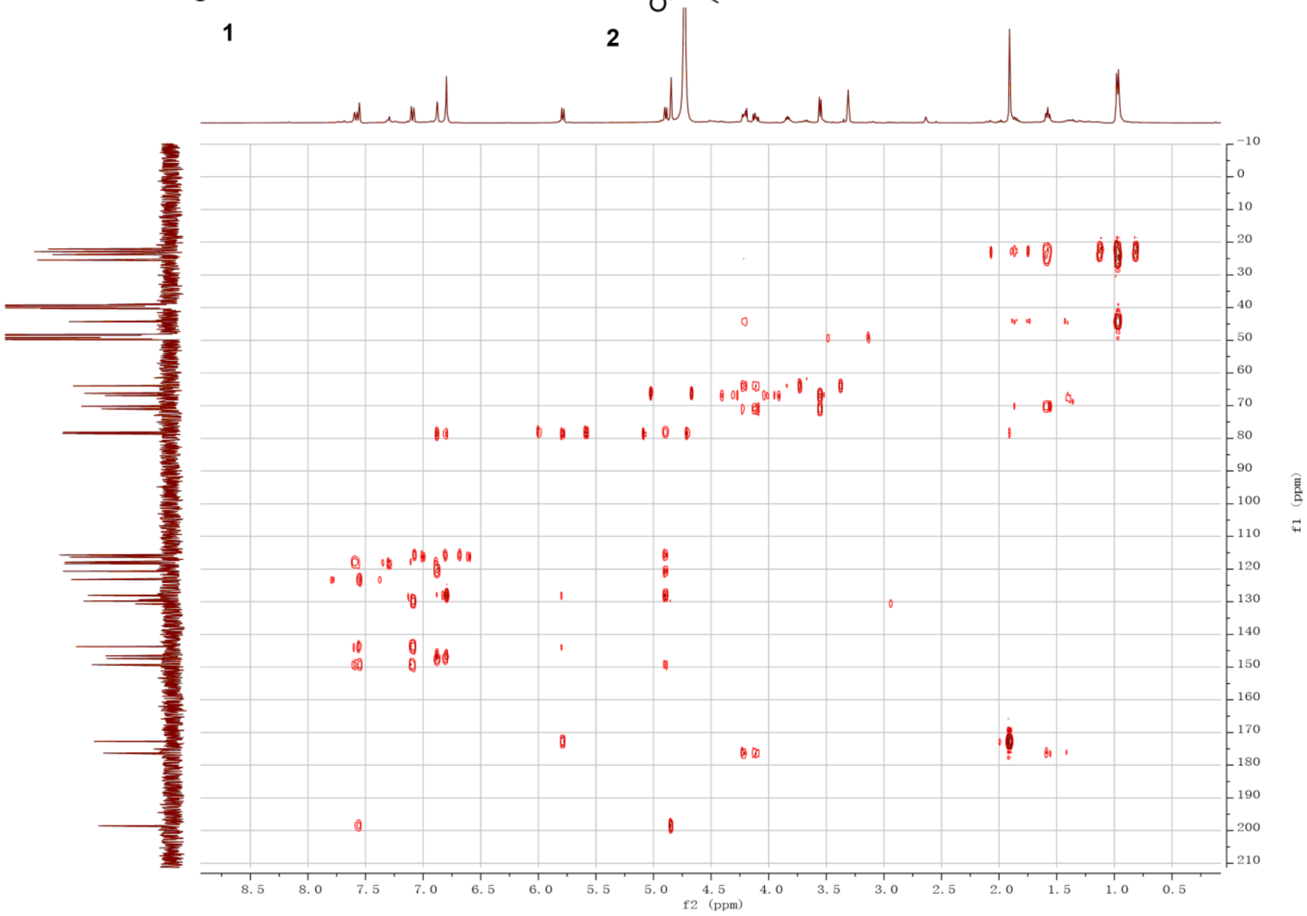

Figure S4. HMBC spectrum ($CDCl_3$, 400 MHz) of **1** and **2**

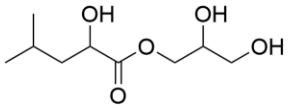

1

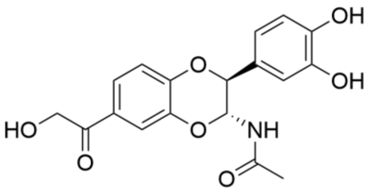

2

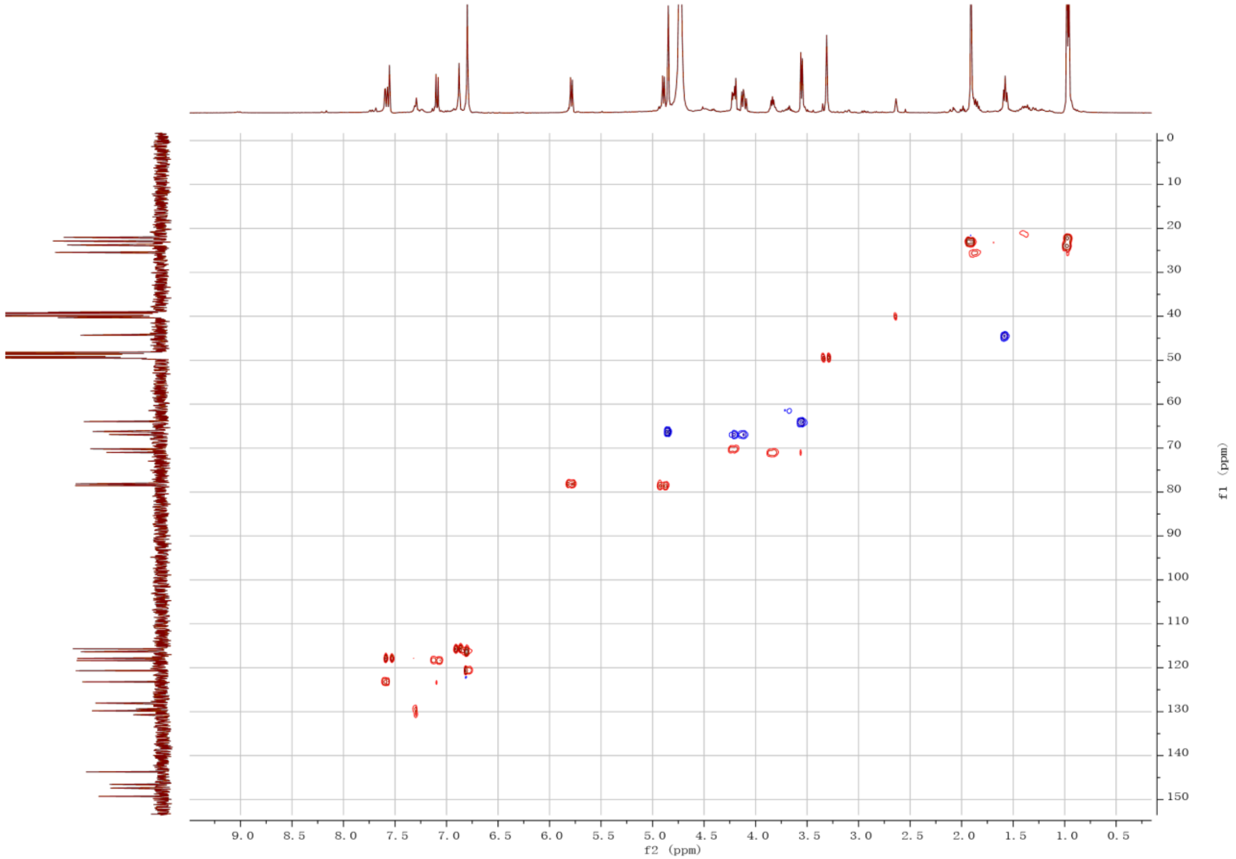

Figure S5. HSQC spectrum ($CDCl_3$, 400 MHz) of **1** and **2**

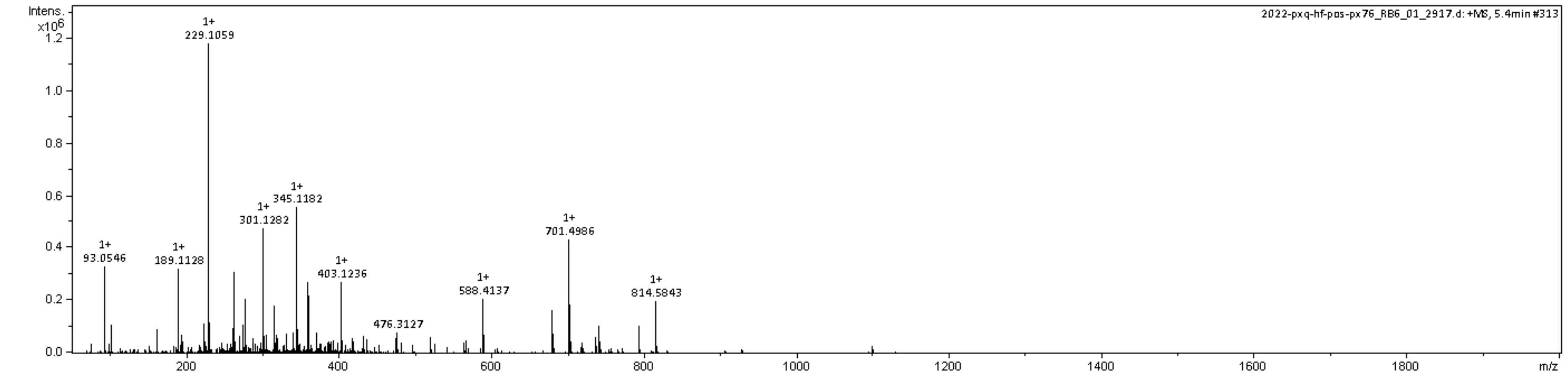

Figure S6. (+)-HR-ESI-MS (positive mode) of **1**

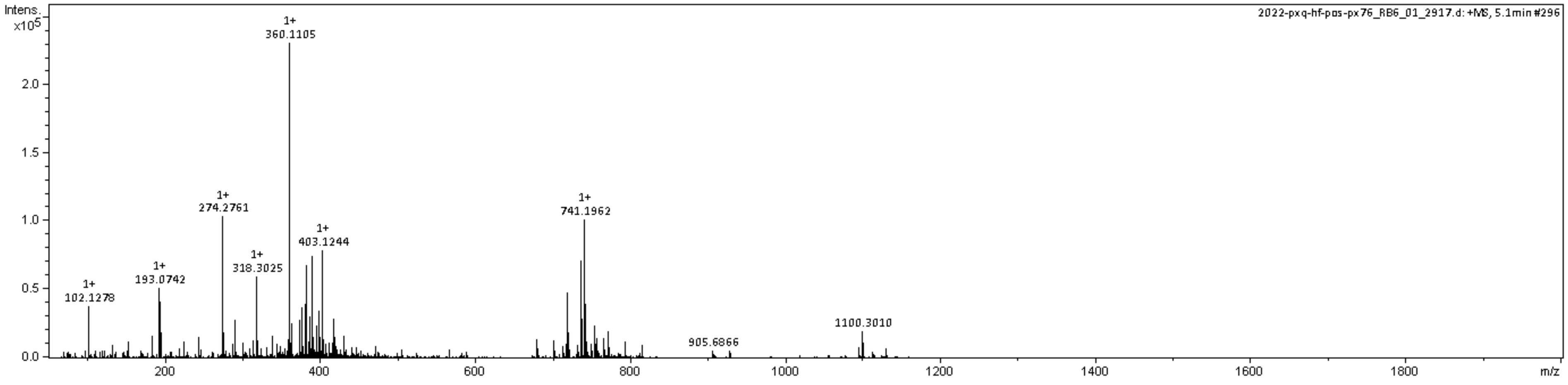

Figure S7. (+)-HR-ESI-MS (positive mode) of **2**

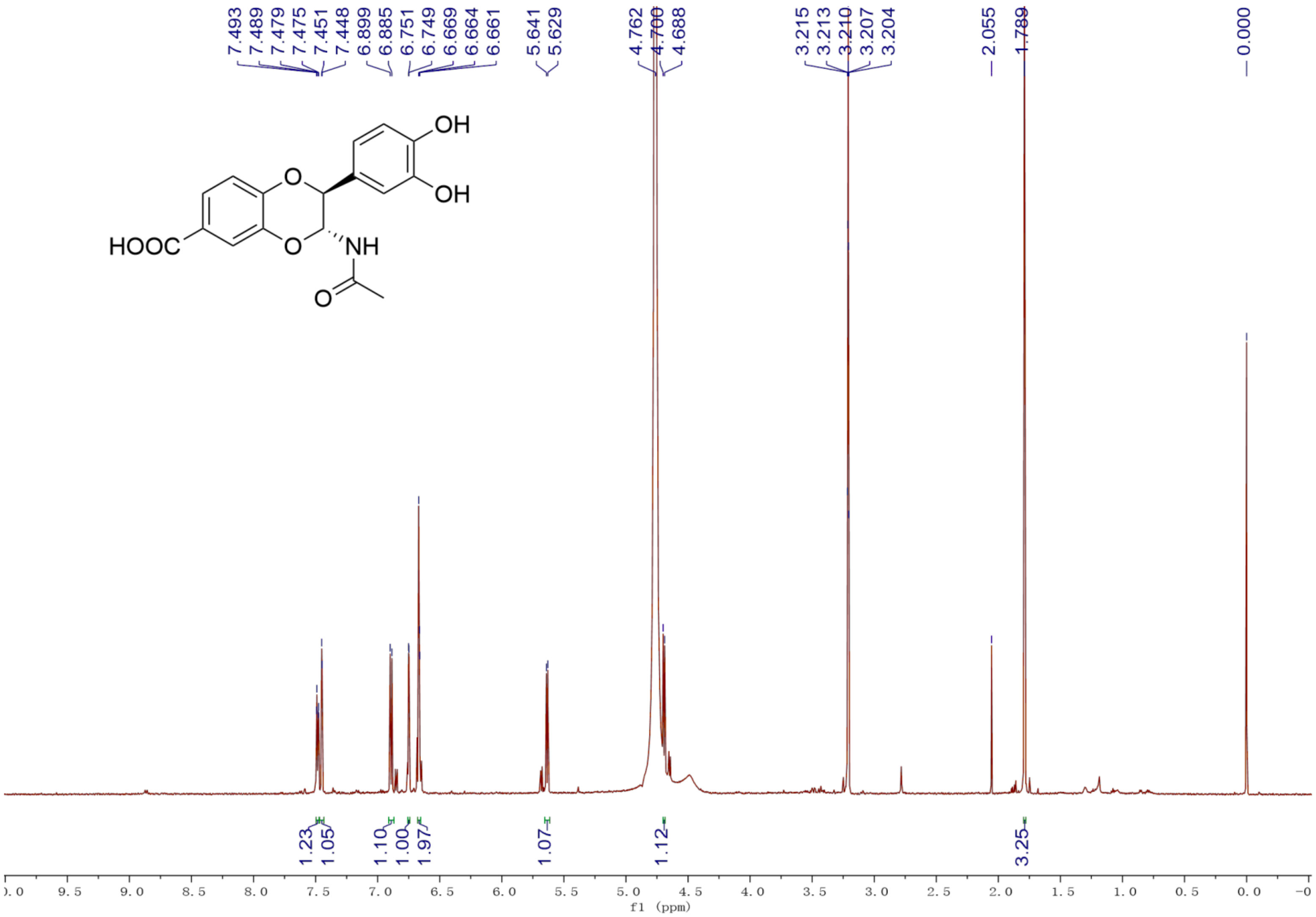

Figure S8. ^{1}H NMR spectrum (MeOD, 600 MHz) of **3**

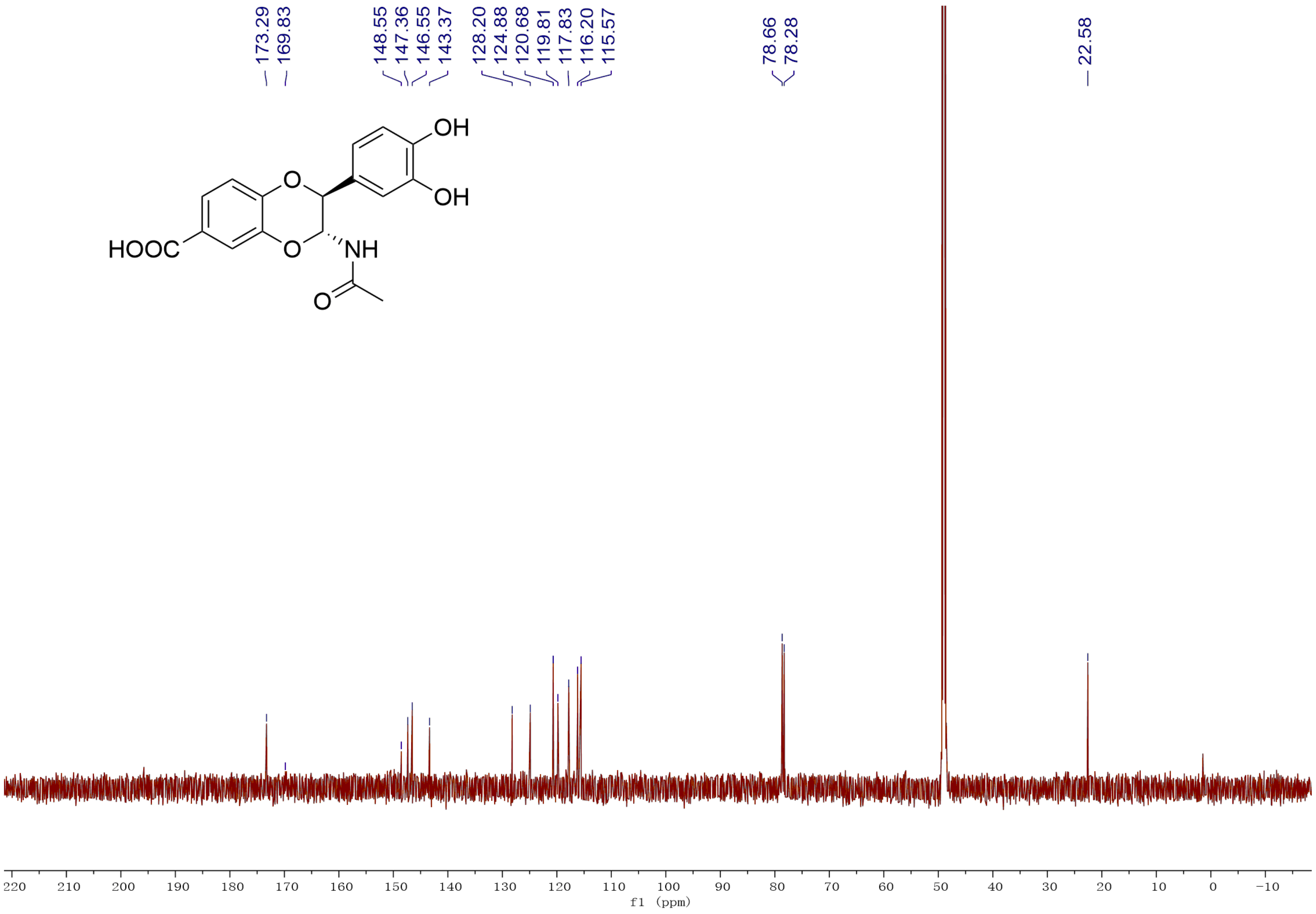

Figure S9. ^{13}C and DEPT spectra (MeOD, 150 MHz) of **3**

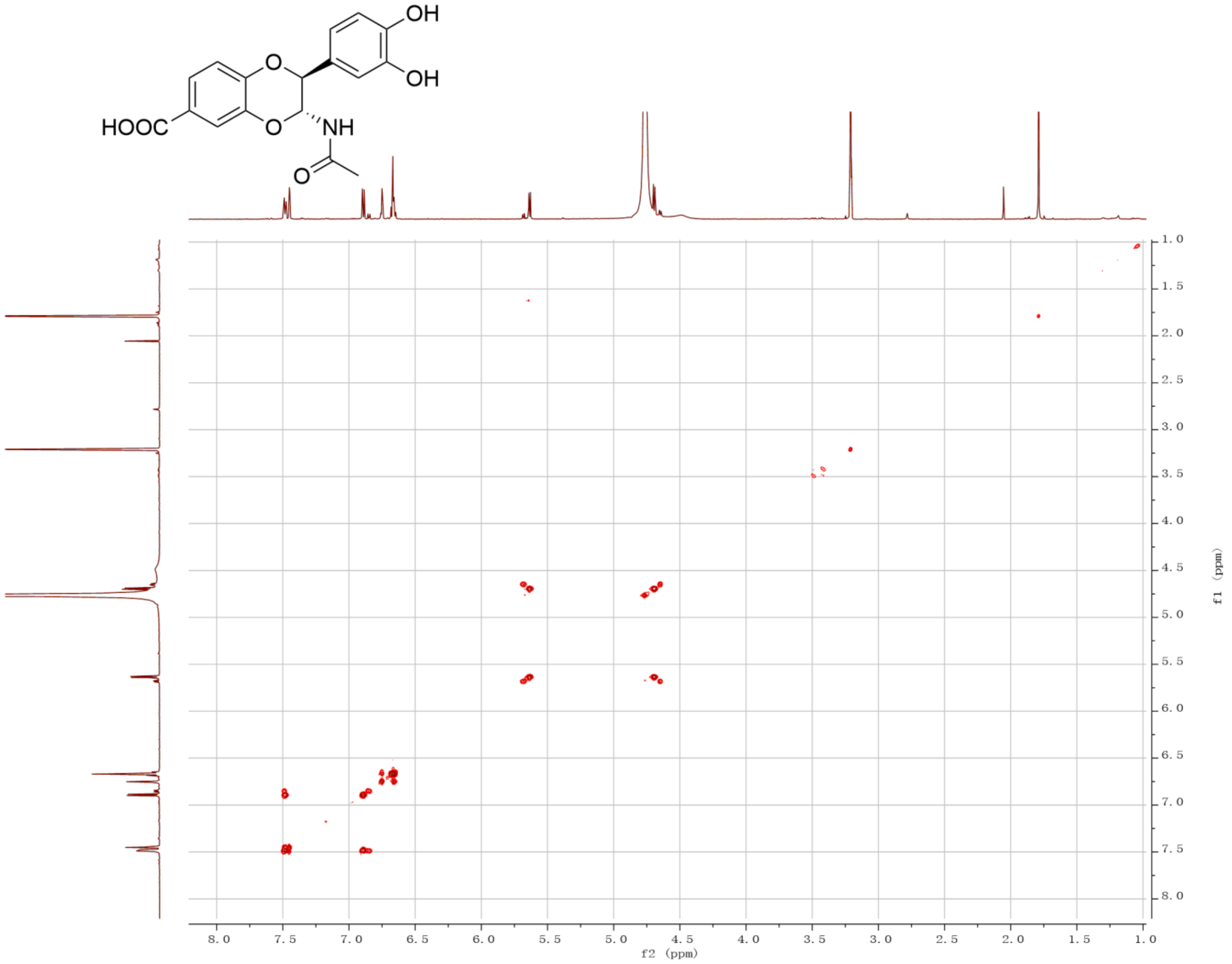

Figure S10. ^{1}H-^{1}H COSY spectrum (MeOD, 600 MHz) of **3**

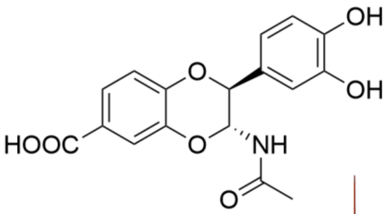

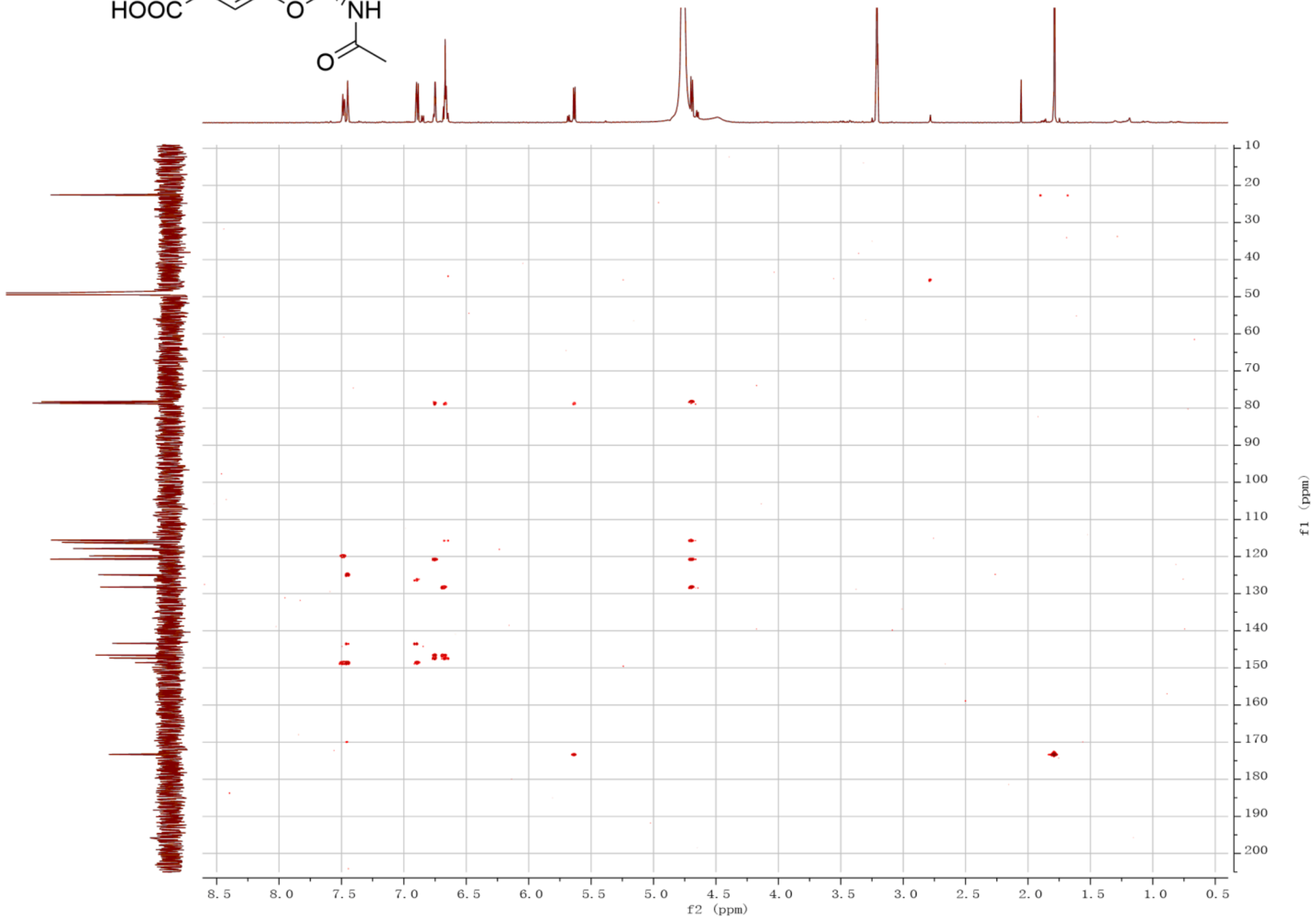

Figure S11. HMBC spectrum (MeOD, 600 MHz) of **3**

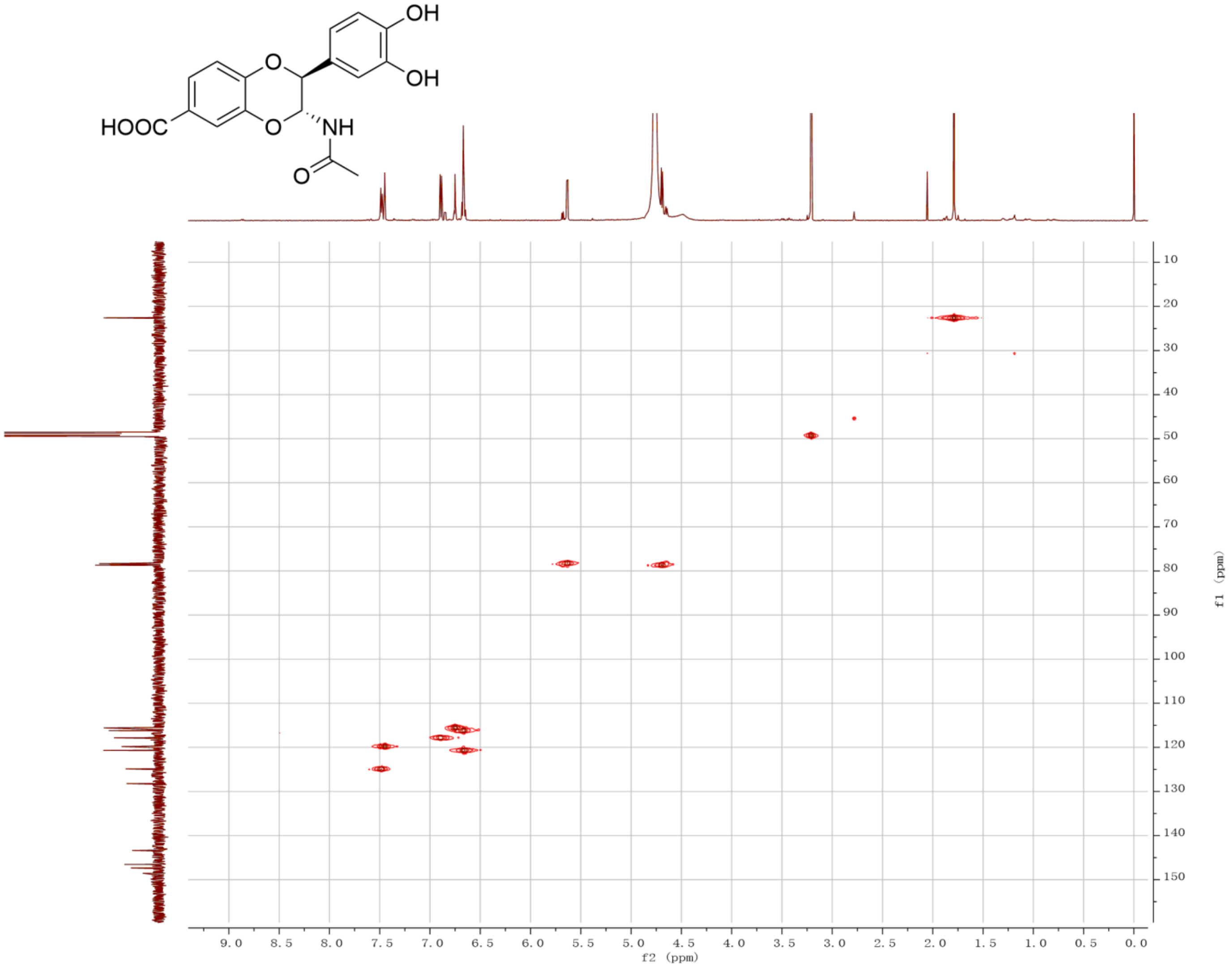

Figure S12. HSQC spectrum (MeOD, 600 MHz) of **3**

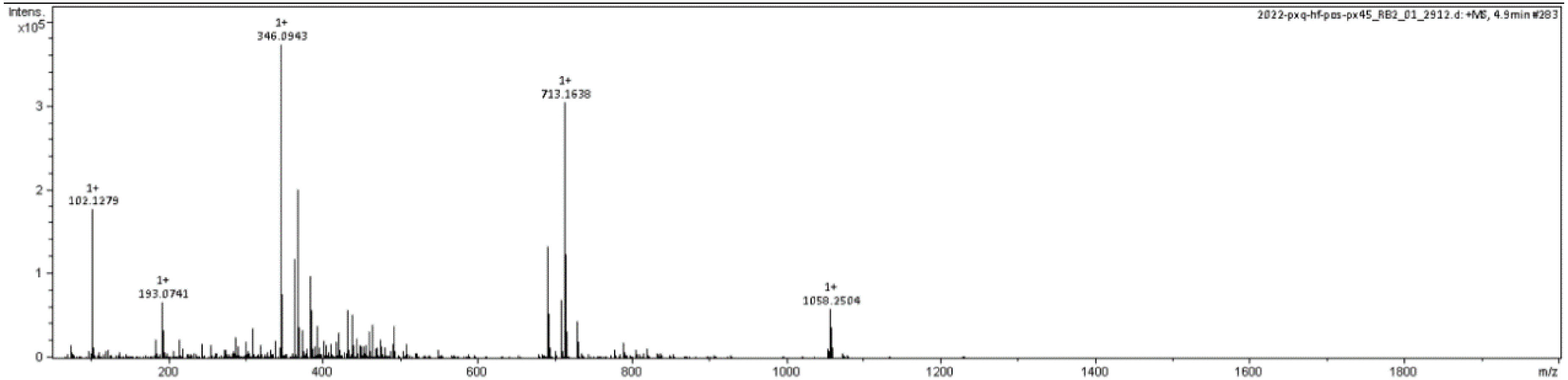

Figure S13. (+)-HR-ESI-MS (positive mode) of **3**.

	Isomer 1	Isomer 2
DP4-H	62.04	37.96
DP4-C	0.42	99.58
DP4-J	16.69	83.31
DP4-HC	0.68	99.32
JDP4full	0.14	99.86

exp	Isomer 1	Isomer 2
78.7	78.5102	79.3502
78.3	82.1111	83.844
143.9	137.5625	137.5239
118	115.8809	116.0103
130	126.3808	125.5244
123.4	118.9686	119.6647
118.5	117.2307	116.4299
149.4	139.4691	141.0555
198.7	191.3518	190.9138
66.4	64.374	64.5418
128.2	138.7384	139.4746
115.9	102.0354	104.72
147.5	151.6526	150.4549
116.5	96.8045	96.1212
146.7	152.1974	151.0201
120.8	103.2944	105.1969
172.9	158.8373	158.811
23	19.5232	22.8919
4.89	4.6186	5.2099
5.79	5.6836	5.7468
7.55	7.8303	7.8014
7.59	7.6189	7.645
7.09	7.3456	7.2284
4.85	4.3031	4.3081
4.85	4.3254	4.3291
6.88	6.4619	6.5242
6.8	5.7965	5.7927
6.8	6.4947	6.4051
1.91	1.9966	1.6655
7.2	2.1704	9.8759

DP4 | Shifts_Unsc | Shifts | Unsc_Errors | Errors

exp	Isomer 1	Isomer 2
78.7	81.6756	81.294
78.3	85.408	86.0424
143.9	142.8848	142.7641
118	120.4112	120.0314
130	131.2946	130.0847
123.4	123.6117	123.8929
118.5	121.8104	120.4748
149.4	144.861	146.4959
198.7	198.6388	199.1793
66.4	67.023	65.6465
128.2	144.1036	144.8253
115.9	106.06	108.1014
147.5	157.4895	156.4279
116.5	100.638	99.0153
146.7	158.0542	157.025
120.8	107.365	108.6053
172.9	164.9367	165.2574
23	20.534	21.6364
4.89	4.8597	5.437
5.79	5.9131	5.9584
7.55	8.0364	7.9535
7.59	7.8273	7.8017
7.09	7.557	7.3971
4.85	4.5696	4.5817
4.85	4.5476	4.5614
6.88	6.6829	6.7133
6.8	6.0247	6.003
6.8	6.7154	6.5977
1.91	2.2662	1.9952
7.2	2.1704	9.8759

DP4 | Shifts_Unsc | Shifts | Unsc_Errors | Errors

Figure S14. ML-JDP4 results of 2*S**,3*S**-**2** (Isomer 1) and 2*S**,3*R**-**2** (Isomer 2).
